# Predicting hormonal deterioration following transsphenoidal surgery for non-functioning pituitary adenomas: A systematic review and meta-analysis

**DOI:** 10.1007/s12020-026-04712-6

**Published:** 2026-08-01

**Authors:** Daniel Ameen, Youssef ElSabban, Dimitrios Kalaitzoglou, Jonathan Shapey, Sinan Barazi, Nicholas Thomas, Simon Aylwin, Eleni Maratos

**Affiliations:** 1 Monash Health, Clayton, Melbourne, Australia; 2 School of Public Health and Preventive Medicine, Monash University, Melbourne, Australia; 3 College of Medicine, Mohamed Bin Rashid University of Medicine and Health sciences, Dubai Health, Dubai, United Arab Emirates; 4 Department of Neurosurgery, King’s College Hospital NHS Foundation Trust, London, UK; 5 Department of Endocrinology, King’s College Hospital NHS Foundation Trust, London, UK

**Keywords:** Non-functioning pituitary adenoma, Transsphenoidal surgery, Hypopituitarism, Endocrine deterioration, Systematic review

## Abstract

**Background:**

Endoscopic transsphenoidal surgery is indicated when non-functioning pituitary adenomas (NFPAs) cause visual compromise, but its timing is less certain in patients with preserved vision and intact pituitary function, where the risk of new post-operative pituitary dysfunction influences decision-making. This systematic review aimed to determine the rate of new post-operative endocrine deficits following transsphenoidal surgery for NFPAs and to identify predictive factors.

**Methods:**

This review followed PRISMA guidelines. Studies reporting predictors of post-operative endocrine deterioration in adults undergoing transsphenoidal surgery for NFPAs were included. Predictors were synthesised narratively, with meta-analysis where at least two studies used comparable definitions. Pooled proportions estimated the overall rate of deterioration. Certainty of evidence was assessed using GRADE.

**Results:**

Eleven studies comprising 2,350 patients were included. The pooled proportion developing new post-operative endocrine deterioration was 16% (95% CI 13–20%; I² = 60%). Larger tumour volume was associated with deterioration, and gross total resection with reduced odds compared with subtotal resection. Cavernous sinus invasion and other predictors showed inconsistent associations, while age, sex, comorbidities, apoplexy, pituitary gland or stalk visibility, surgeon experience and surgical approach were not associated with risk. Overall certainty of evidence was low to very low.

**Conclusion:**

New post-operative endocrine deficits affect approximately one in six patients after transsphenoidal surgery for NFPAs. Tumour volume was the most consistent predictor of endocrine deterioration, suggesting a potential role for earlier intervention in selected patients, although evidence certainty was low to very low. Standardised reporting and prospective studies are needed to guide risk stratification decision-making.

**Supplementary Information:**

The online version contains supplementary material available at 10.1007/s12020-026-04712-6.

## Introduction

Non-functioning pituitary adenomas (NFPAs) are among the most common pituitary lesions, accounting for up to 54% of surgically treated pituitary adenomas [1]. These tumours typically present with symptoms related to mass effect, including visual disturbance, and are frequently associated with new-onset hypopituitarism [2].

When surgical management is indicated, a transsphenoidal approach remains the gold standard for resection [3, 4]. The primary objectives of surgery in this cohort are relief of mass effect and maximal safe tumour resection, while preserving normal pituitary function [5, 6]. While surgery is clearly indicated in patients with visual compromise, its role in asymptomatic patients is less well defined [7]. This uncertainty reflects the risks of transsphenoidal surgery, which include causing new pituitary dysfunction [5].

Despite widespread use of transsphenoidal surgery, the true incidence of post-operative pituitary dysfunction remains uncertain, in part due to heterogeneity in how post-operative endocrine deterioration is defined and reported across studies [8]. Predictive factors are similarly poorly established, with prior studies reporting inconsistent outcomes and non-uniform predictor definitions [9]. Identifying determinants of post-operative endocrine outcomes is therefore a key priority, as this information is critical for appropriate patient selection, informed pre-operative counselling, and optimisation of post-operative surveillance strategies.

The current study systematically reviewed the predictors of new post-operative endocrine deterioration following transsphenoidal resection of non-functioning pituitary adenomas. In addition, the reported rates of post-operative endocrine deterioration across the existing literature were assessed.

## Methods

**Fig. 1:**
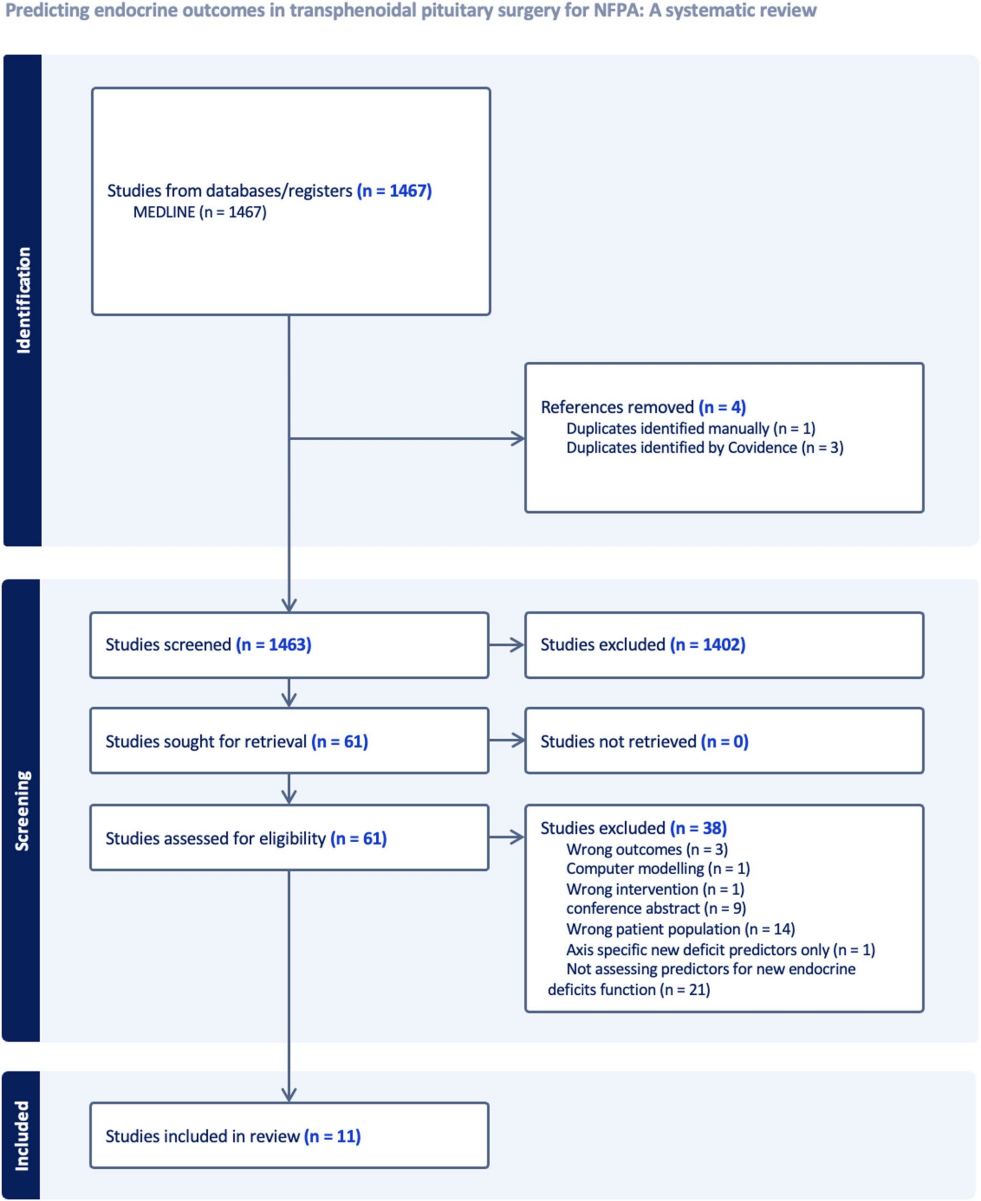
**–** PRISMA flow diagram for study inclusion

The Preferred Reporting Items for Systematic Reviews (PRISMA) 2020 checklist and flow diagram were used as the eligibility and inclusion criteria during the search and selection process (See Fig. 1). This review was prospectively registered on the Prospective Register of Systematic Reviews (PROSPERO), identified under CRD420251139616.

### Search strategy

Studies were identified by searching multiple databases, including Ovid Medline, Embase and the Cochrane Library from database inception to May 2025 was conducted. A systematic search was conducted using MeSH terms and keywords related to non-functioning pituitary adenomas, endocrine outcomes, surgery and predictive/prognostic factors. The full search strategy is provided in Supplementary Table 1.

Studies were initially screened independently by two reviewers (D.A and Y.E), and full-text version of the relevant studies were obtained and assessed against the inclusion criteria. Disagreements were settled through discussion among reviewers (D.A and Y.E). A third party was adjudicated for any disagreement.

### Inclusion and exclusion criteria

Inclusion criteria were any primary studies that included patients with non-functioning pituitary adenomas (NFPA) undergoing transsphenoidal resection (either endoscopic or microscopic methods). For inclusion, the primary study must have reported specifically on at least one predictor of new post-operative endocrine deterioration following resection and have reported on post-operative endocrine outcomes. For the purposes of this review, “new post-operative endocrine deterioration” encompasses both patients with normal pre-operative pituitary function who develop a new deficit post-operatively, and those with pre-existing hypopituitarism who develop an additional axis deficit following surgery.

Studies were excluded if they focused exclusively on functioning pituitary adenomas or if the predictors for functioning as compared to the non-functioning adenoma group could not be differentiated. Studies were excluded if the study did not specifically analyse predictors of new endocrine deficits/hormone deterioration. In cases where studies assessed predictors of post-operative pituitary function, instead of assessing new post-operative hormone deterioration, they were excluded. Case reports, narrative reviews, editorials and conference abstracts were excluded.

### Data extraction

Study information was extracted and included the following variables: author, year of publication, study design setting and sample size. For endocrine outcomes, overall rates of new endocrine deficits/hormone deterioration were recorded, with the definitions of hormone deterioration noted.

Predictive factors assessed in each study were collated. Predictor definition, direction of effect and statistical significance was extracted from each study. The reported effect measures (ORs, HRs, mean differences) with 95% confidence intervals were extracted. Where possible, the raw data were extracted and effect sizes manually calculated.

### Risk of bias assessment and certainty of evidence

#### Risk of bias assessment

All included studies were critically appraised by two independent reviewers for methodological quality in the review using the JBI SUMARI critical appraisal instruments for cohort studies. Any disagreements that arose were resolved through discussion. The certainty of evidence was assessed as per the Grading of Recommendations Assessment, Development and Evaluation (GRADE) approach.

### Data synthesis and statistical analysis

#### Narrative synthesis

The primary outcome was a composite outcome of the development of worsening pituitary function after surgery.

A structured narrative synthesis was performed for all predictors, grouped as follows: patient-related factors, tumour-related factors and surgical/technical factors. Predictors that were assessed in a single study only or reported with non-comparable definitions amongst different studies were synthesised narratively.

#### Statistical analysis

A meta-analysis was conducted when $\:\ge\:$ 2 studies reported a predictor and outcome in a comparable manner. Binary outcomes were synthesised using pooled odds ratios (ORs) with 95% confidence intervals after having collected the number of patients with pituitary deterioration vs. preserved function in the predictor and control groups. Continuous predictors were synthesised using unstandardised mean differences, assuming unequal group variances after having collected the mean, standard deviation and sample size in the predictor and the control groups. Finally, pooled proportions were calculated to estimate the overall rate of endocrine deterioration amongst the included studies. Measures of heterogeneity used where I^2^. Data was analysed in December 2025. Analyses were conducted using SPSS software V29 for the predictors, and STATA v19 for pooled proportions. Statistical tests were two-tailed with significance set to *P* < 0.05.

## Results

As shown in Fig. 1, the initial search retrieved a total of 1467 records. After screening, 11 total studies met the inclusion criteria [10–20].

### Characteristics of included studies

The characteristics of included studies are summarised in Table 1. The 11 studies which met the inclusion criteria were published between 2015 and 2022 and comprised a total of 2350 patients undergoing transsphenoidal surgery for non-functioning pituitary adenomas. As shown in Table 1 all studies were observational, including nine retrospective cohort studies and two prospective cohort studies.

**Table 1:** – Characteristics of included studies

Study (Author, Year)	Country	Study design	Sample size (*n*)	Surgical approach (microscopic, endoscopic, both, NA)	Predictors assessed	Rate of new postoperative deficits * n/*N* (%)
Araujo-Castro 2022	Spain	Multicentre Retrospective Cohort	146	Endoscopic	Age; sex; preoperative visual impairment; hyperprolactinaemia; comorbidities; tumour size; cavernous sinus invasion; apoplexy; tumour consistency; extent of resection; surgeon experience; CSF leak; previous surgery	11/82 (13.4%)
Biamonte 2021	Italy	Single-centre Retrospective Cohort	160	Endoscopic	Age, sex, cavernous sinus invasion, tumour diameter	19/156 (12.2%)
Chinezu 2017	France	Retrospective Cohort	307	Endoscopic	Age	7/33 (21%) *
Gondim 2015	Brazil	Single-centre Retrospective Cohort	374	Endoscopic	Age	63/374 (16.8%)
Jahangiri 2016	USA	Single-centre Retrospective Cohort	282	Both	Age, sex, tumour size, surgical resection extent, preoperative gland volume	42/305 (13.7%)
Little 2020	USA	Multicentre Prospective Cohort	177	Both	Age, sex, BMI, tumour diameter, tumour volume, cavernous sinus invasion, prior surgery, surgical resection goal, EOR	14/145 (9.7%)
Little 2020 **	USA	Multicentre Prospective Cohort	171	Both	Surgical resection goal	As above
Palpan-Flores 2021	Spain	Single-centre Retrospective Cohort	100	Endoscopic	Surgeon experience/learning curve, Volumetric extent of resection	17/100 (17%)
Seejore 2021	UK	Single-centre retrospective cohort	173	Endoscopic	Extent of resection, surgeon experience/learning curve	25/83 (30.1%) *
Song 2022	China	Single-centre Retrospective Cohort	514	Both	Microscopic vs. endoscopic	10.4% **
Vivancos Sanchez 2021	Spain	Single-centre Retrospective Cohort	117	Endoscopic	Age, sex, tumour volume, cavernous sinus invasion, pituitary gland visibility on MRI, stalk visibility on MRI, extent of resection	19/96 (19.8%)

* These studies only reported the rate of endocrine deficits in patients who were normal preoperatively (i.e. did not include those who had one or more pituitary deficits preoperatively, but experienced an additional deficit) – these studies were not included in the pooled proportion meta-analysis

** Averaged if only axis specific rates were reported – these were not included in the pooled proportion meta-analysis

### Risk of bias and certainty of evidence

#### Quality appraisal of included studies

Critical appraisal of eleven cohort studies examining surgical outcomes in non-functioning pituitary adenomas using the JBI checklist revealed generally high methodological quality with consistent limitations as shown in Supplementary Table 2. All studies demonstrated valid and reliable measurement of exposures (Q3: 11/11) and outcomes (Q7: 11/11), although the method of outcome determination differed by study. Studies had adequate follow-up duration (Q8: 11/11), and appropriate statistical analyses (Q11: 11/11). No study (Q6 0/11) required patients to be free from pre-existing endocrine deficits pre-operatively, reflecting the inclusive definition of new post-operative deterioration used across the included studies. Most studies identified relevant confounding factors (Q4: 9/11, with only Little 2020a and 2020b rated “no”), though fewer employed explicit strategies to address confounding (Q5: only 4/11 “yes” - Araujo-Castro, Biamonte, Jahangiri, Vivancos; 7/11 “no”). Six comparative studies appropriately recruited similar groups from the same population (Q1 and Q2: 6/11 “yes”; 4/11 “not applicable” for single-cohort descriptive studies). The most notable weakness across the evidence base was incomplete reporting of follow-up completeness and attrition, with only three studies clearly documenting complete follow-up (Q9: Gondim, Jahangiri, Little 2020b) and only one explicitly describing strategies to address incomplete follow-up (Q10: Gondim only). Eight studies were rated “unclear” for follow-up completeness (Q9), representing a significant methodological reporting gap that limits assessment of potential attrition bias across the majority of included studies.

The method of endocrine assessment varied across included studies. The majority used standard basal hormone measurements (serum cortisol, IGF-1, free T4, TSH, LH, FSH, testosterone or oestradiol, and prolactin) supplemented by dynamic stimulation tests where indicated (most commonly short Synacthen test or insulin tolerance test for corticotroph axis assessment). Thresholds for defining deficiency were not uniformly reported and may have differed between institutions and over time. This methodological heterogeneity in how endocrine status was ascertained represents an important limitation and is discussed further in the Limitations section.

#### Certainty of evidence

Overall, the quality of the included studies was low to very low (Table 2) as guided by the GRADE approach.

### Pooled proportion of postoperative endocrine deterioration

**Fig. 2:**
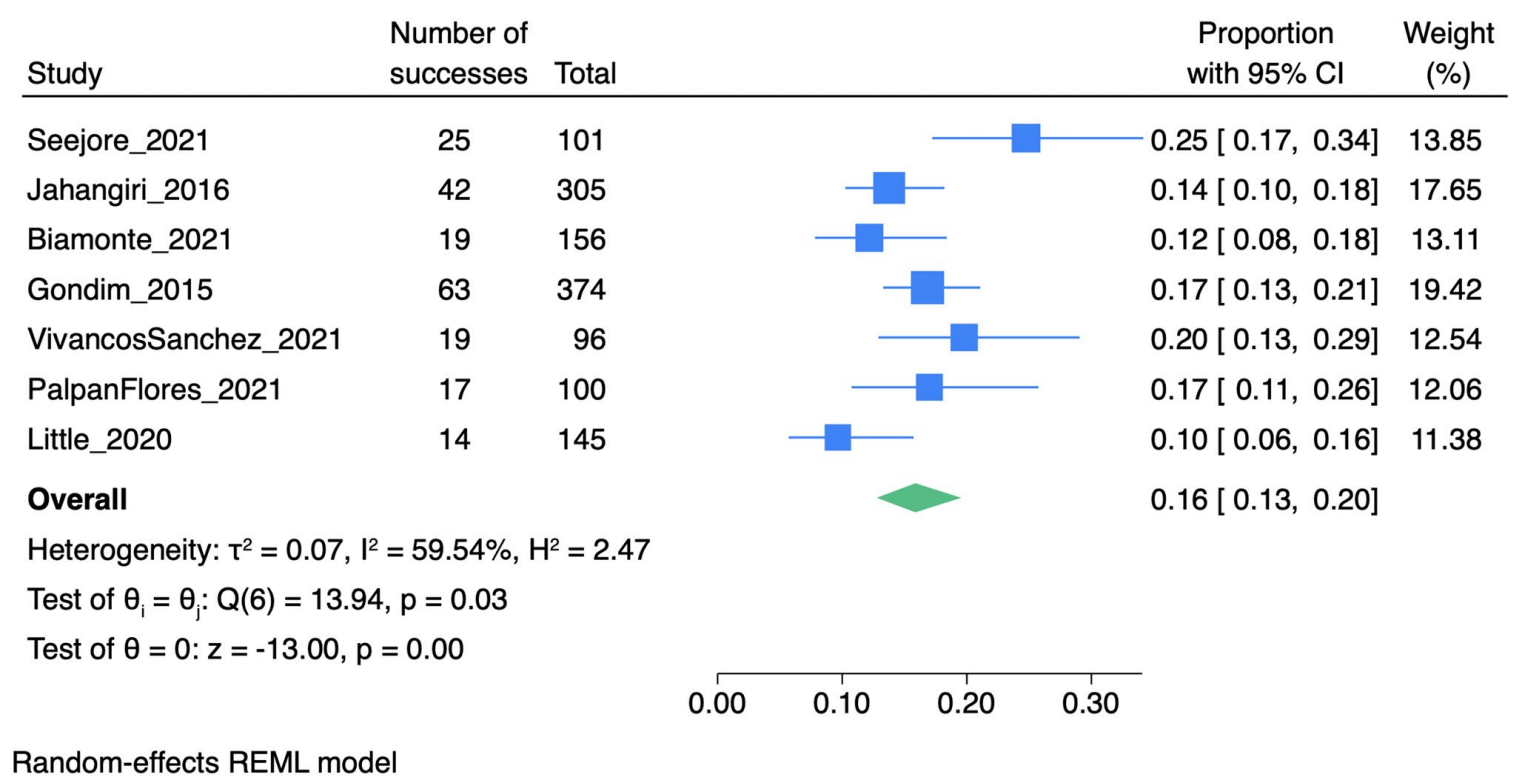
Meta-analysis of pooled proportions for endocrine deterioration following transsphenoidal surgery

Seven studies [11, 13, 14, 16–18, 20] reported the proportion of patients who developed new or worsening post-operative pituitary deficits following surgery for non-functioning pituitary adenomas and were included in the pooled proportion meta-analysis (Fig. 2). Four additional studies were excluded from the pooled analysis because of inconsistent outcome reporting, most commonly due to the use of non-comparable denominators (e.g. reporting rates only among patients with preserved pre-operative pituitary function or reporting axis-specific rates only), which precluded meaningful quantitative analysis. Among the included studies, reported rates of post-operative endocrine deterioration varied from 10% to 25%.

**Fig. 3:**
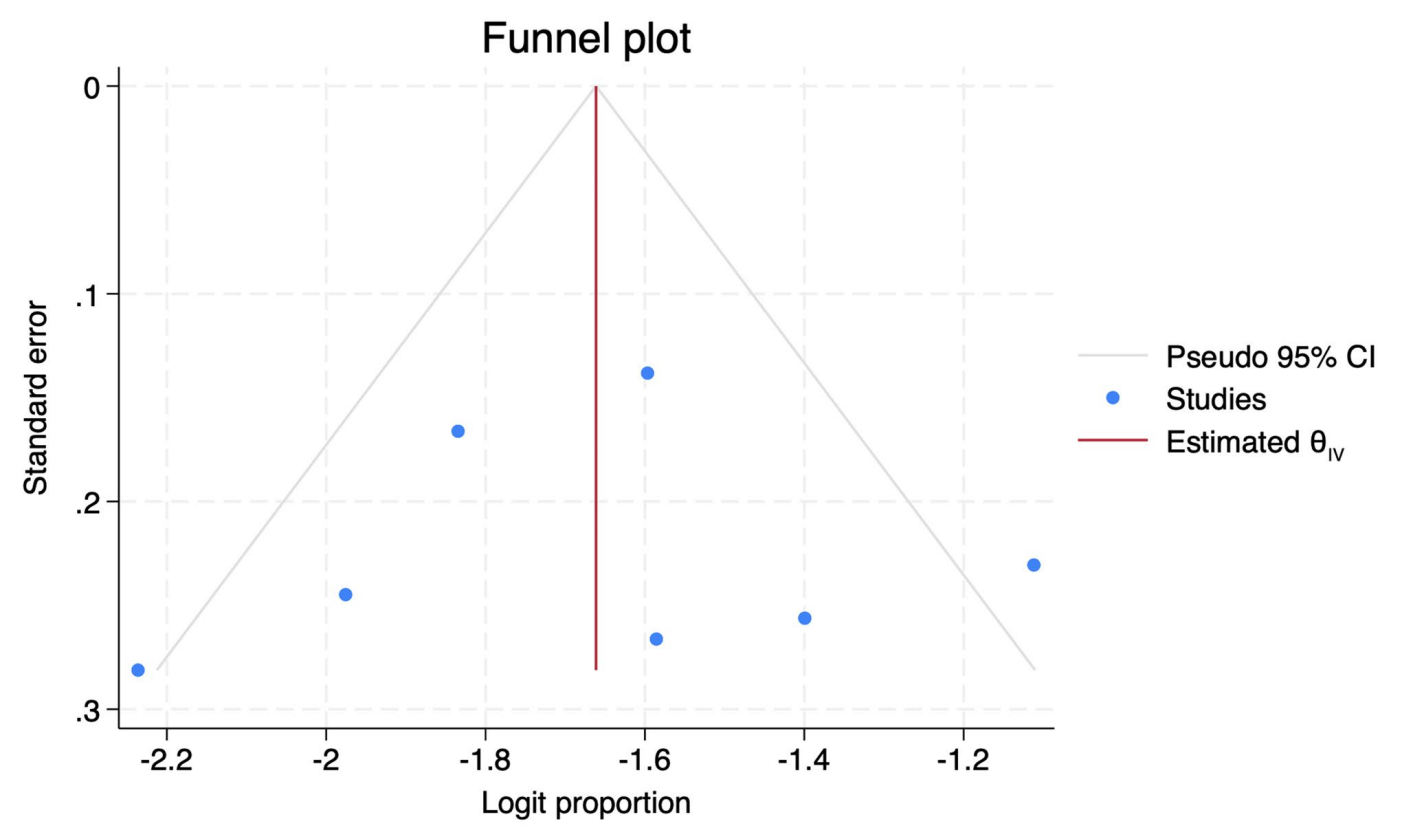
Funnel plot for pooled proportions of patients with post-operative endocrine deterioration showing no publication bias

Using a random-effects model, the pooled proportion of patients developing new post-operative endocrine deficits was 16% (95% CI 13–20%). There was moderate heterogeneity between studies (τ² = 0.07; I² = 59.5%), indicating variability in reported rates beyond chance alone. In addition, the funnel plot shown in Fig. 3 demonstrates symmetry, indicating no publication bias.

### Predictors of any new pituitary deficit

Predictors for the development of worsening pituitary function were analysed. Table 2 shows the predictors that were assessed and summarises the overall significance of each predictor, based on narrative synthesis or meta-analysis.

**Table 2:** – Predictors assessed for worsening endocrine deficits following transsphenoidal surgery

Predictor	Number of Studies	Key Findings	Statistical Finding	Meta-analysis Performed	GRADE (certainty of evidence)
**Patient Factors**					
Age	7 (Chinezu 2017; Gondim 2015; Little 2020; Araujo-Castro 2022; Vivancos Sanchez 2021; Jahangiri 2016; Biamonte 2021)	Across all studies, age was not statistically associated with new pituitary deficits	MESE = 0.07; 95% CI − 0.34 to 0.48; *p* = 0.74; I² = 0%	Yes (2 studies only)	⊕⊕⊝⊝ LOW
Sex	4 (Little 2020; Araujo-Castro 2022; Jahangiri 2016; Biamonte 2021)	No significant relationship found in the meta-analysis and in individual studies	Pooled OR = 0.68; 95% CI 0.29–1.68; *p* = 0.38	Yes (2 studies)	⊕⊝⊝⊝ VERY LOW
BMI	1 (Little 2020)	No significant relationship	*p* = 0.82	No	
Preoperative visual impairment	1 (Araujo-Castro 2022)	No significant relationship	Adjusted OR = 5.7; *p* = 0.065	No	
Preoperative hyperprolactinaemia	1 (Araujo-Castro 2022)	No significant relationship	*p* = 0.509	No	
Comorbidities	1 (Araujo-Castro 2022)	No significant relationship	*p* > 0.05 for all comorbidities	No	
**Tumour Characteristics**					
Size (diameter)	3 (Jahangiri 2016; Biamonte 2021; Araujo-Castro 2022)	No significant difference	*p* > 0.05	No	⊕⊕⊝⊝ LOW
Volume	2 (Little 2020; Vivancos Sanchez 2021)	Significant relationship - Larger tumours associated with new/worsened deficits	Mean difference = 4.3 mL; 95% CI 0.76–7.85; *p* = 0.02	Yes	⊕⊝⊝⊝ VERY LOW
Clinical apoplexy	1 (Araujo-Castro)	No significant relationship	*p* = 0.42	No	
Cavernous sinus invasion	4 (Little; Araujo-Castro; Vivancos Sanchez; Biamonte 2021)	Mixed results – mostly non-significant relationship on Meta-analysis	pooled OR 1.99, 95% CI (0.67–5.86), *p* = 0.21	Yes	⊕⊝⊝⊝ VERY LOW
Tumour consistency	1 (Araujo-Castro)	No significant relationship	*p* = 0.937	No	
Pituitary stalk visibility	1 (Vivancos Sanchez)	No significant relationship	All *p* > 0.05	No	
Pituitary gland visibility	1 (Vivancos Sanchez)	No significant relationship	Adjusted OR = 2.47; 95% CI 0.67–9.05; *p* = 0.173	No	
**Surgical/Technical Factors**					
Surgeon experience	4 (Seejore; Little; Araujo-Castro; Palpes Flores)	No significant relationship	Pooled OR 1.60, 95% CI 0.53–4.85, *p* = 0.40	Yes	⊕⊝⊝⊝ VERY LOW
Extent of resection	4 (Seejore; Little; Vivancos Sanchez; Araujo-Castro 2022)	Significant relationship in favour of GTR showing reduced odds of new pituitary deficits.	GTR showed pooled OR = 0.49; 95% CI 0.24–0.98; *p* = 0.04; I² = 0%	Yes	⊕⊕⊝⊝ LOW
Surgical goal	1 (Little 2020)	No significant relationship	*p* = 0.32	No	
Previous surgery	1 (Araujo-Castro)	No significant relationship	p-0.651	No	
CSF leak	1 (Araujo-Castro)	Significantly association with CSF leak for increased odds of a new pituitary deficit	2.8% in patients with preserved function versus 18.2% in those with new deficits, *p* = 0.028	No	
Microscopic vs. Endoscopic approach	1 (Song)	No significant relationship	*P* = 0.228	No	

## Patient factors

### Age

**Fig. 4:**
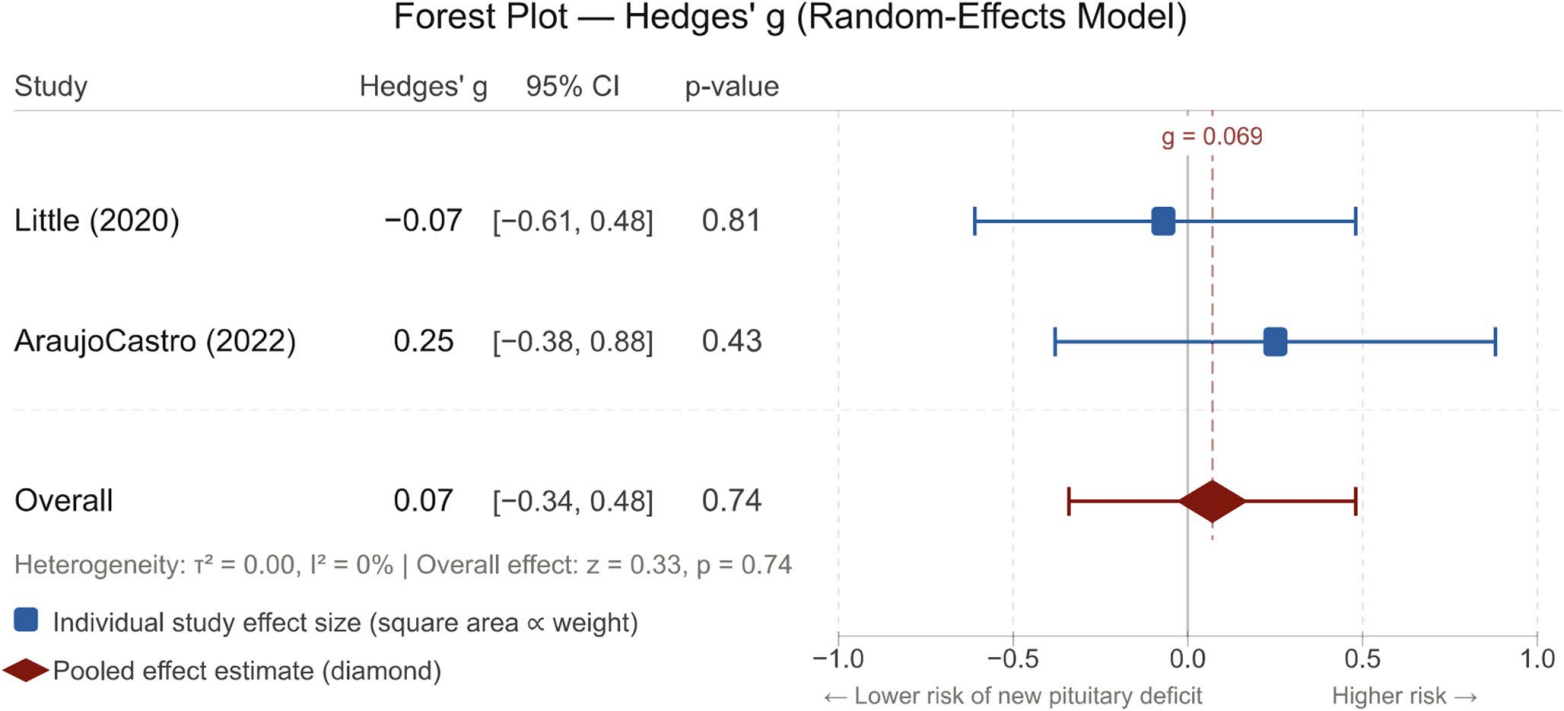
Forest plot of age (continuous variable) as a predictor of new postoperative endocrine deficits following transsphenoidal surgery for NFPA’s

Seven studies investigated age as a potential predictor of post-operative endocrine deterioration, and none demonstrated a statistically significant association [10–14, 16, 20]. Two studies [10, 16] analysed age as a continuous variable in relation to the development of new or worsened post-operative endocrine deficits. As shown in Fig. 4, a pooled analysis of these two studies found no association between age and postoperative endocrine deterioration (MESE = 0.07; 95% CI − 0.34 to 0.48; *p* = 0.74), with no evidence of heterogeneity (I² = 0%). These findings were consistent with the remaining 5 studies [11–14, 20], which used heterogeneous categorical age cut-offs or reported their results narratively, that all reported no statistically significant association with post-operative endocrine deterioration.

### Sex

**Fig. 5:**
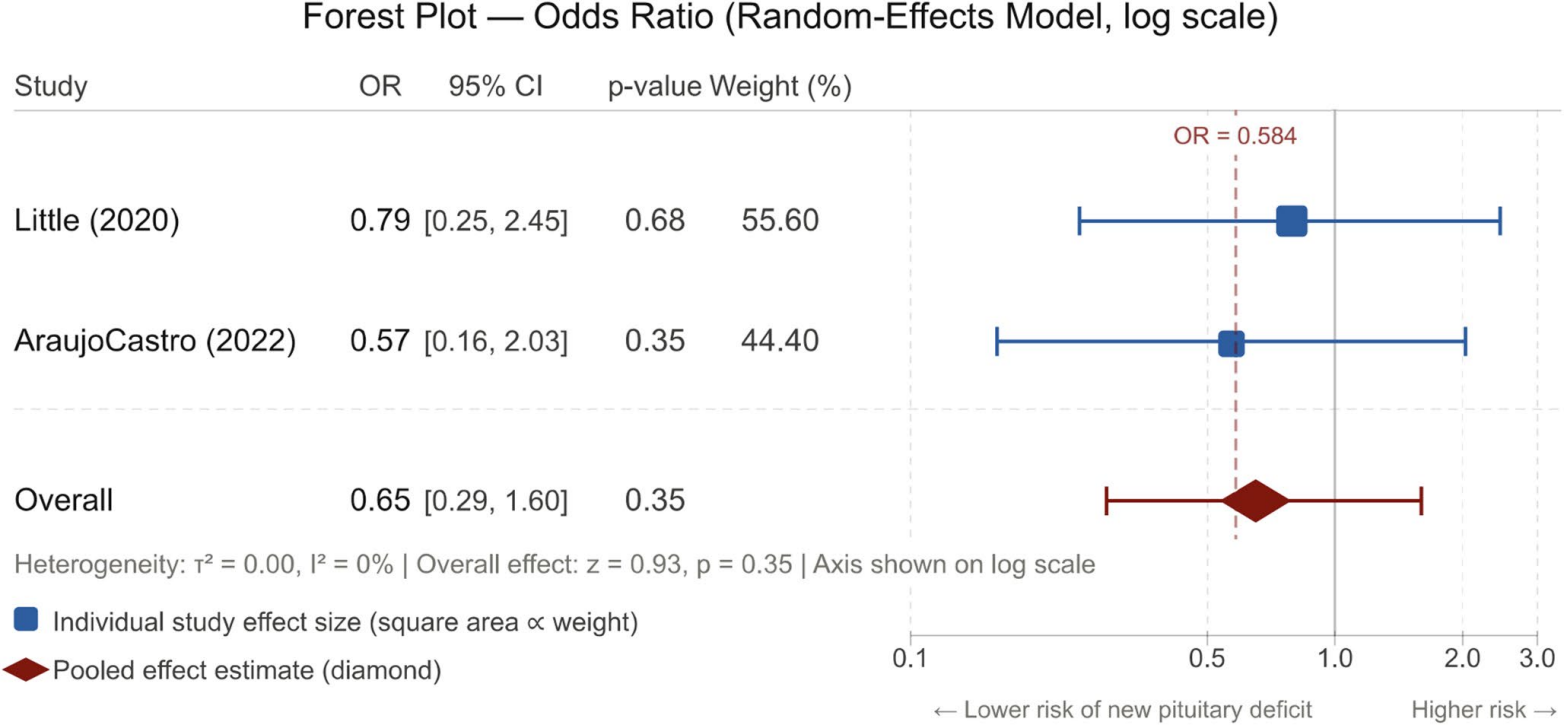
– Forest plot of sex with pooled Odds Ratio’s a predictor of postoperative endocrine deterioration. * Being female was treated as the “Exposure” in the meta-analysis

Four studies evaluated sex as a potential predictor of post-operative endocrine deterioration [10, 11, 14, 16]. As shown in Fig. 5, in a pooled analysis of two studies [10, 16], sex was not statistically significantly associated with the development of new pituitary deficits (pooled OR 0.68; 95% CI 0.29–1.68; *p* = 0.38). This finding aligns with the remaining two studies [11, 14], which also reported no significant association between sex and the occurrence of new post-operative pituitary deficits.

### BMI

One study [16] examined the association between BMI and the development of new post-operative pituitary deficits. No statistically significant relationship was found (*p* = 0.82).

### Preop visual impairment

One study [10] evaluated the association between pre-operative visual impairment and post-operative endocrine deterioration. After adjustment for tumour size, there was no statistically significant association indicating increased odds (adjusted OR 5.7; *p* = 0.065).

### Preoperative hyperprolactinaemia

One study evaluated the association between pre-operative hyperprolactinaemia and post-operative endocrine deterioration [10]. This showed that pre-operative hyperprolactinaemia was not associated with the development of new anterior pituitary deficits, occurring in 16.9% of patients with preserved post-operative pituitary function compared with 9.1% of patients who developed new deficits (*p* = 0.509).

### Comorbidities

No statistically significant association was found in any of the assessed comorbidities (hypertension, obesity, heart disease, diabetes) (*p* > 0.05 for all) [10].

## Tumour characteristics

### Tumour Size

Three studies examined tumour diameter as a predictor of post-operative endocrine deterioration [10, 11, 14]. None of these studies detected a statistically significant difference in tumour size. A meta-analysis was not performed due to heterogeneous categorisation of tumour size across studies.

### Tumour volume

**Fig. 6:**
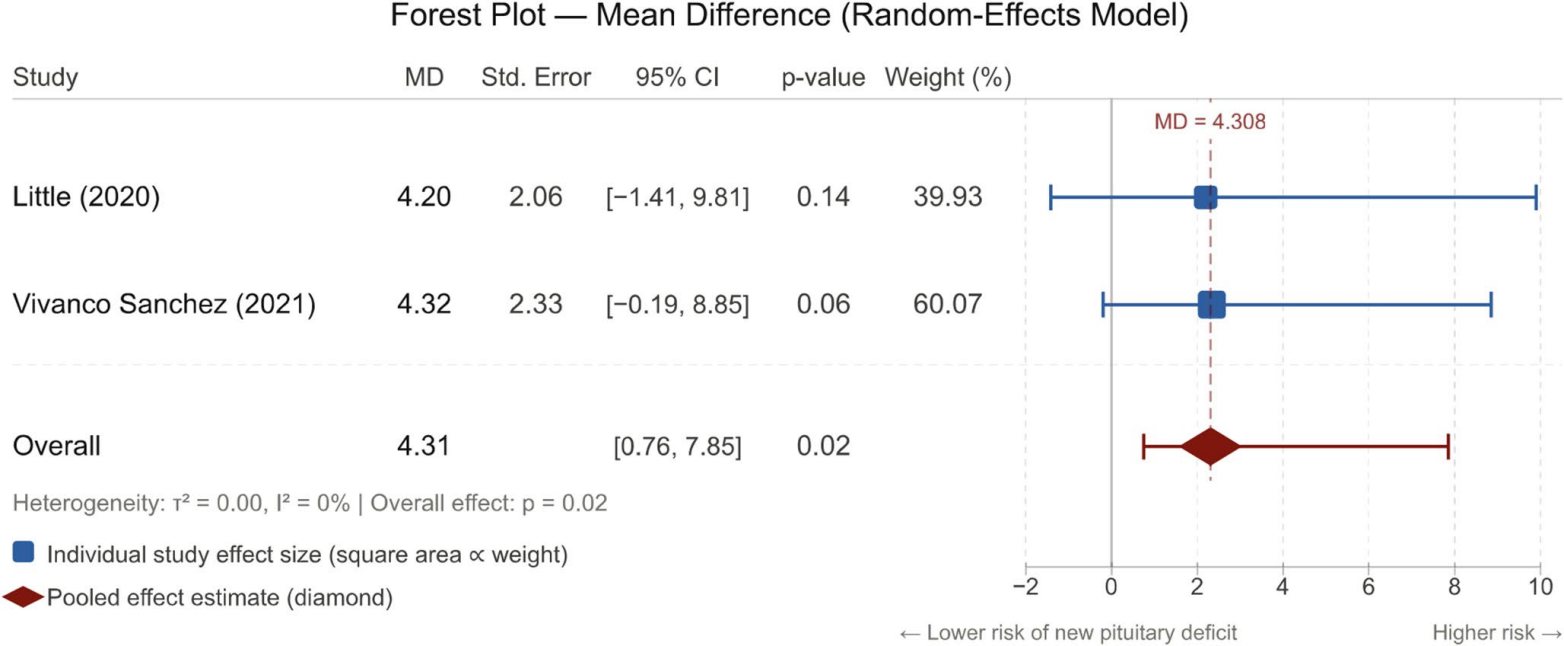
– Forest plot of tumour volume as a predictor for postoperative endocrine deterioration

Two studies evaluated tumour volume as a predictor of post-operative endocrine deterioration [16, 20]. In the pooled analysis, patients who developed new or worsened post-operative endocrine deficits had a statistically significantly larger baseline tumour volumes, with a mean difference of 4.3 mL (95% CI 0.76–7.85; *p* = 0.02) (See Fig. 6). There was no evidence of heterogeneity between the studies (I² = 0%).

### Pituitary apoplexy

One study evaluated the association between pituitary apoplexy and the development of new or worsening anterior pituitary deficits [10]. No statistically significant relationship was observed when comparing patients with preserved pituitary function (5.6%) to those with new deficits (0%) (*p* = 0.42).

### Cavernous sinus invasion

**Fig. 7:**
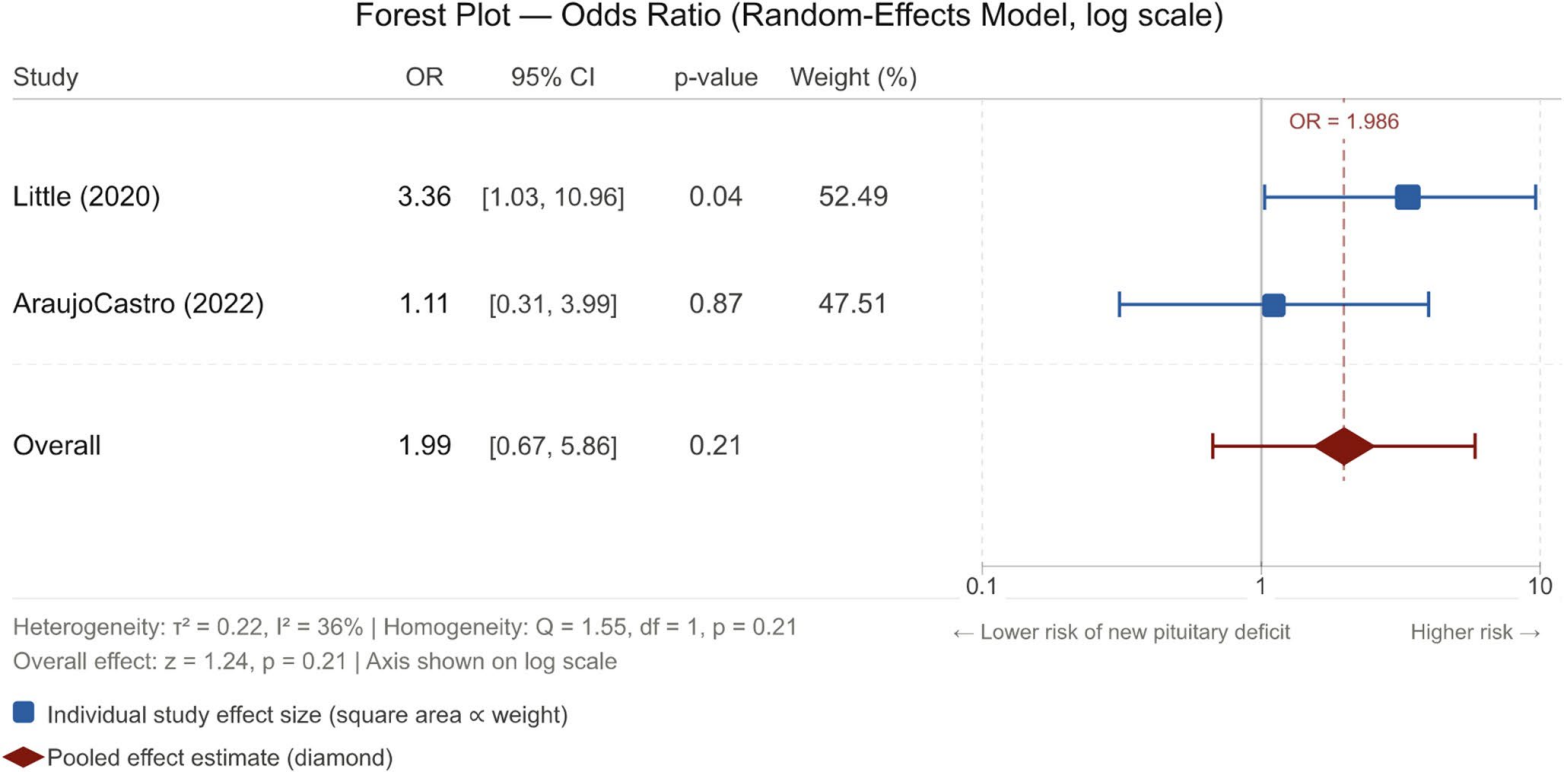
– Forest plot of cavernous sinus invasion (Knosp grade 3–4 vs. 0–2) showing pooled odds ratios for endocrine deterioration

Four studies evaluated cavernous sinus invasion, defined as Knosp grade 3–4 versus 0–2, as a predictor of post-operative endocrine deterioration [10, 11, 16, 20]. In a pooled analysis of two studies that reported raw data, cavernous sinus invasion (Knosp > 2) was associated with higher odds of endocrine deterioration (pooled OR 1.99, 95% CI 0.67–5.86), although this did not reach statistical significance (*p* = 0.21) (See Fig. 7). Heterogeneity was low to moderate (I² = 36%). One study demonstrated a significant association, while the other showed a similar direction of effect but with wide confidence intervals. The results of this pooled analysis were concordant with one study that narratively reported results, showing no statistically significant association (*p* > 0.05) between Knosp grade and hormone function postoperatively [20]. However, the other study showed contradicting result with a statistically significant increased Hazard ratio (HR) for presurgical cavernous sinus invasion (HR 4.19, 95% CI 1.39–12.66; *p* = 0.01) [11].

### Tumour consistency

One study examined the association between “hard consistency” and the development of new or worsening pituitary deficits [10]. No statistically significant relationship was found, with similar proportions in patients with preserved function (31.3%) and those with new deficits (30.0%) (*p* = 0.937).

### Pituitary stalk visibility

One study evaluated pituitary stalk visibility on MRI as a potential predictor of new endocrine deficits [20]. Pre-operative and post-operative stalk visibility was not associated with post-operative endocrine improvement or deterioration, including the development of new anterior pituitary deficits at 12 months (all *p* > 0.05).

### Pituitary gland visibility

One study assessed pituitary gland visibility on MRI as a predictor of postoperative pituitary deterioration [20]. The adjusted OR for having a non-visible gland was 2.47 (95% CI, 0.67–9.05; *p* = 0.173), indicating no statistically significant association.

## Surgical/technical factors

### Surgeon experience/learning curve (early vs. late experience)

**Fig. 8:**
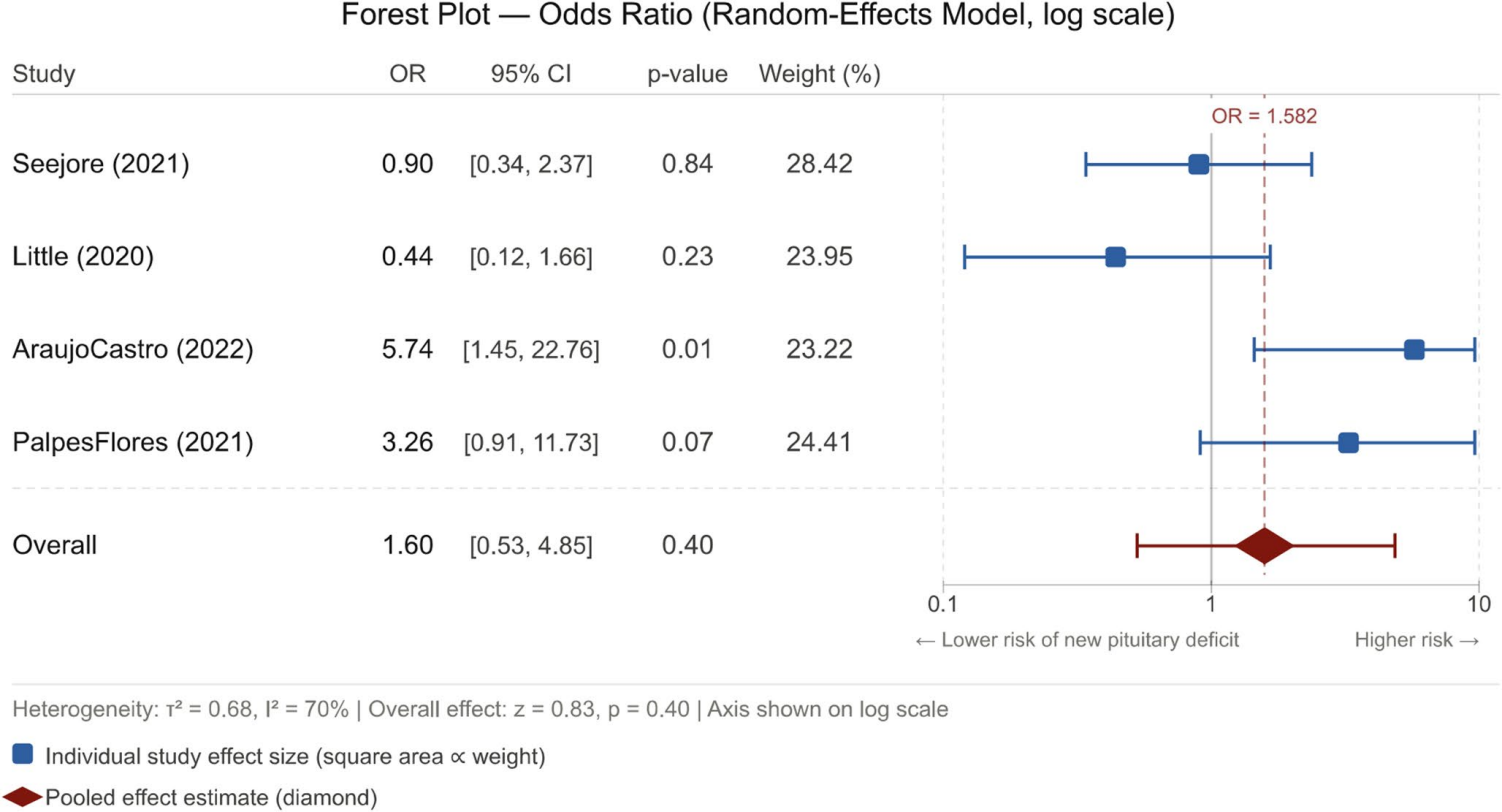
Forest plot of surgeon experience (early vs. late learning curve) and risk of new postoperative endocrine deficits following transsphenoidal surgery for non-functioning pituitary adenomas. *Early stage of learning curve is treated as the exposure

Four studies assessed surgeon learning curve as a predictor of post-operative endocrine deterioration, using heterogeneous definitions of surgical experience [10, 16–18]. In a pooled analysis, earlier learning curve was associated with higher odds of endocrine deterioration, although this did not reach statistical significance (pooled OR 1.60, 95% CI 0.53–4.85, *p* = 0.40) (See Fig. 8). There was substantial heterogeneity between studies (I² = 70%), likely reflecting differences in how learning curve was defined and measured across studies.

### Extent of resection

**Fig. 9:**
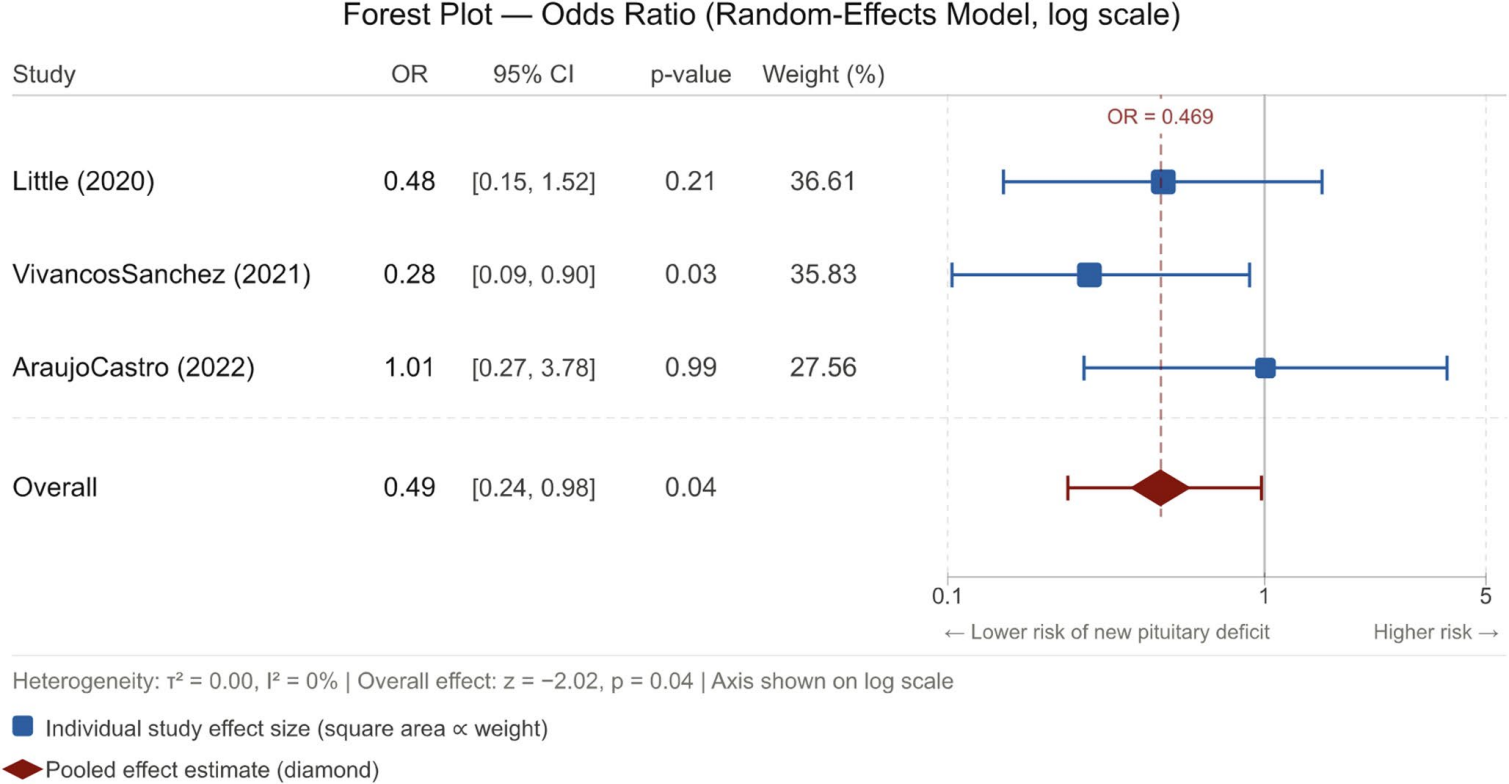
Forest plot for extent of resection (Gross total resection being assessed) as a predictor for endocrine deterioration

Four studies assessed the extent of resection as a predictor of worsening endocrine outcomes post-operatively [10, 16, 18, 20]. Three of these studies contributed to a pooled analysis (Fig. 9), which demonstrated that gross total resection (GTR) was associated with significantly lower odds of developing new or worsened post-operative endocrine deficits compared with subtotal resection (STR) (OR 0.49; 95% CI 0.24–0.98; *p* = 0.04) [10, 16, 20]. There was low heterogeneity between studies (I² = 0%). The remaining study that was not included in the meta-analysis reported that patients with new axis deficits had a mean extent of resection (EOR) of 78.6%, compared with 73.8% in those with preserved pituitary function; this difference was not statistically significant on unpaired t-test (*p* = 0.37) .

### Surgical goal

One study evaluated the association between surgical goal and the development of new pituitary deficits [15]. No significant relationship was observed at 6 months, with new deficiencies occurring in 11.8% of patients after gross total resection (GTR: 20 of 170) compared to 17.3% after subtotal resection (STR: 8 of 46) (*p* = 0.32).

### Previous surgery

One study assessed previous surgery as a predictor of new deficits [10]. This showed no statistically significant association with an OR of 0.61 (95%CI 0.14–2.60), *p* = 0651.

### CSF leak

Postoperative cerebrospinal fluid (CSF) leak was evaluated as a potential predictor of endocrine deterioration in one study [10]. A significant association was found between post-operative CSF leak and the development of new or worsened anterior pituitary deficits, with rates of 2.8% in patients with preserved function versus 18.2% in those with new deficits (*p* = 0.028).

## Discussion

### Summary of principal findings

In this systematic review, new post-operative endocrine deficits occurred in approximately one in six patients (16%, 95% CI 13–20%) following transsphenoidal surgery for non-functioning pituitary adenomas. Overall, tumour volume—and intraoperative factors reflecting surgical complexity such as CSF leak – were more strongly associated with endocrine deterioration than patient demographics or surgeon-related factors.

### Interpretation of pooled rates and comparison with existing literature

In pooled analysis of 1,277 patients, the rate of new post-operative endocrine deterioration was 16%, consistent with previously reported ranges of 0–36.6% in the literature [8]. This estimate should be interpreted cautiously, as only studies that clearly defined new post-operative deterioration were included, incorporating both patients with normal baseline function who developed new deficits and those with pre-existing hypopituitarism who experienced further decline (as defined in the Introduction). Importantly, these deterioration rates should be considered alongside post-operative recovery rates. Prior studies have reported endocrine recovery in 29–55% of patients, depending on which axis is assessed and the timing of those assessments. This suggests that, in selected patients, the likelihood of hormonal improvement may exceed the risk of new deficits [21, 22, 6]. 

Interpretation of these rates is limited by variation in how endocrine deterioration was defined and when post-operative assessment occurred across studies. Most studies used basal pituitary hormone panels (with variable use of dynamic testing, particularly for the corticotroph axis), and assessments were performed at differing postoperative time points, while cohorts often included both patients with normal baseline function and those with pre-existing hypopituitarism who experienced further deterioration, limiting direct comparison and preventing formal synthesis of baseline endocrine risk.

### Positive predictors of endocrine deterioration

Tumour volume emerged as the most consistent predictor of post-operative endocrine deterioration, with patients who developed new deficits having significantly larger tumours (mean difference 4.3 mL). In contrast, diameter-based size measures were not consistently associated with endocrine outcomes, likely reflecting heterogeneous reporting and categorical thresholds across studies. This finding highlights the limitations of one-dimensional tumour metrics and supports volumetric assessment as a more sensitive and clinically relevant measure of endocrine risk. This observation is consistent with the hypothesis that patients with NFPAs demonstrating progressive volumetric growth on serial imaging may warrant consideration of earlier surgical intervention. However, this inference rests on only two studies and was graded as very low certainty evidence; it should therefore be regarded as hypothesis-generating rather than a basis for altering current practice.

Interestingly, gross total resection (GTR) was associated with a reduced risk of new endocrine deficits compared with subtotal resection. This association likely reflects favourable tumour characteristics—such as smaller size and invasiveness, and clearer tumour–gland interfaces—rather than a direct protective effect of complete resection. Tumour consistency is an important correlate of surgical difficulty: soft tumours generally permit more complete and atraumatic resection, whereas fibrous or calcified tumours require more forceful dissection and are more likely to result in subtotal removal with greater gland manipulation. Although formal meta-analysis of tumour consistency was not possible due to single-study reporting, one included study found no significant independent association between hard consistency and endocrine deterioration; however, this may reflect insufficient statistical power rather than a true null effect. The finding that GTR is associated with lower endocrine morbidity most plausibly reflects this complex confounding by tumour texture, size, and invasiveness—all of which simultaneously determine the feasibility of complete resection and the risk of gland injury. Surgical intent to pursue GTR did not increase endocrine risk, suggesting that appropriate surgical goals do not compromise pituitary function when tumour anatomy permits safe dissection [23]. Post-operative CSF leak was associated with increased endocrine deterioration in one study; however, this finding was not adjusted for tumour size and likely reflects confounding by surgical complexity [24]. Importantly, CSF leak is a post-operative complication and, as such, cannot serve as a pre-operative risk-stratification tool. Its association with endocrine deterioration most plausibly reflects confounding by surgical complexity and tumour characteristics, rather than a direct causal relationship. CSF leak should therefore be understood as a marker of operative difficulty and not be incorporated into pre-operative counselling frameworks as an independent predictor.

### Interpretation of null and inconsistent findings

Across included studies, patient demographic factors—including age, sex, and comorbidity burden—were not associated with post-operative endocrine deterioration. Similarly, surgical factors such as surgeon experience, learning curve, surgical approach, and planned extent of resection did not independently influence endocrine outcomes. The absence of a learning-curve effect is reassuring and likely reflects modern pituitary surgery being delivered within multidisciplinary, standardised care pathways with structured supervision and perioperative endocrine protocols. This finding aligns with prior systematic reviews that similarly failed to demonstrate a learning-curve effect for endocrine outcomes in NFPAs [25]. Tumour-related factors such as apoplexy were also not associated with new endocrine deficits, possibly reflecting a ceiling effect due to high rates of preoperative hypopituitarism and the potential stabilising effect of surgical decompression It is worth noting, however, that pituitary apoplexy is strongly associated with larger tumour size, and large tumours undergoing apoplexy are highly likely to have disrupted endocrine function already at presentation. The absence of a significant association in the included study may therefore be partly attributable to a floor effect, wherein a high baseline rate of pre-existing hypopituitarism in apoplextic tumours limits the measurable additional post-operative deterioration [26, 27].

### Limitations

This review is limited by heterogeneity in the definition and reporting of endocrine outcomes. Studies differed in how new post-operative deficits were defined, in baseline patient inclusion, and in criteria used to classify post-operative deficiency, limiting comparability and precluding meta-analysis for several predictors. In addition, non-uniform categorical thresholds for key variables (e.g. tumour size and age) further restricted cross-study synthesis. Most included studies were retrospective, with inconsistent multivariable adjustment, introducing potential selection bias, unmeasured confounding, and limiting causal inference. In addition, Axis-specific predictors were not examined, as this review focused on predictors of any new postoperative pituitary deficit. Furthermore, the methodology used to ascertain endocrine status differed across studies and was not uniformly reported. Included studies generally used standard basal pituitary hormone panels, with dynamic stimulation testing employed variably for corticotroph axis assessment; however, specific assays, cut-off thresholds, and testing protocols were not consistently detailed. This limits the comparability of endocrine outcomes across studies and may have introduced misclassification bias in the reported rates of deterioration. Most importantly, the overall certainty of the evidence was low to very low across all predictors when assessed using the GRADE approach, reflecting the predominantly retrospective, observational nature of the included studies, the small number of studies contributing to each pooled estimate, and the methodological limitations described above. The pooled estimates and predictor associations reported here should therefore be interpreted as the best available synthesis of an inherently limited evidence base rather than as definitive risk estimates, and any clinical inferences—including those relating to the timing of surgery—must be regarded with corresponding caution. Adequately powered, prospective cohorts and carefully curated, standardised databases with pre-specified outcome definitions and consistent multivariable adjustment are needed before these findings can be translated into firm clinical recommendations.

### Clinical implications

From a clinical perspective, our findings have important implications for pre-operative counselling and surgical decision-making. Patients should be informed that the overall risk of new endocrine deterioration following NFPA surgery is modest and largely driven by tumour characteristics rather than patient age or surgeon experience. Additionally, this review supports a nuanced approach to surgical timing in patients with NFPAs. Larger tumour volume was the most consistent predictor of post-operative endocrine deterioration, suggesting that progressive volumetric growth on serial imaging may represent a window of increasing endocrine risk. In this context, surgical intervention prior to the development of significant tumour enlargement or mass effect may be reasonable in selected patients with demonstrated tumour growth, with the aim of limiting exposure to higher tumour volumes and reducing the risk of postoperative hormonal compromise. This consideration may be particularly relevant when counselling patients with radiological progression but minimal or absent visual symptoms. Finally, the relatively low rate of deterioration compared with reported recovery rates may support hypopituitarism as a relative indication for surgery in appropriately selected cases, although this was not the focus of the current systematic review.

### Implications for future research

Future studies should prioritise standardised definitions of endocrine outcomes, consistent reporting of denominators, and volumetric tumour measurements to improve comparability. Large, prospective cohorts with robust multivariable adjustment are needed to better delineate independent predictors and guide personalised surgical decision-making. Future studies should also report the severity of post-operative hypopituitarism, specifying whether patients developed single-axis or multi-axis deficits, or pan-hypopituitarism. Understanding the extent of hormonal loss—not merely its occurrence—is critical for quantifying the true clinical burden and for guiding replacement therapy decisions. Prospective axis-specific reporting with pre-specified, validated biochemical criteria for each pituitary hormone axis (including dynamic testing protocols) would substantially improve the comparability and clinical utility of future studies in this field. These considerations are derived from evidence of low to very low certainty and are intended to inform shared, individualised decision-making rather than to define new surgical thresholds; they require confirmation in prospective studies before being incorporated into routine practice.

## Conclusion

In conclusion, new post-operative endocrine deficits occur in approximately one in six patients undergoing transsphenoidal surgery for non-functioning pituitary adenomas. Among the factors assessed, tumour volume emerged as the most consistent predictor of endocrine deterioration, while patient demographics and surgeon-related factors were not independently associated with risk. These associations were, however, derived from predominantly retrospective studies and were graded as low to very low certainty. The finding that progressive volumetric tumour growth may represent an increasing endocrine risk is therefore best regarded as hypothesis-generating; while it raises the possibility that earlier surgical intervention could be considered in selected patients, this requires confirmation before influencing practice. Adequately powered, prospective studies with standardised endocrine outcome reporting and volumetric tumour assessment are required to refine risk stratification and guide individualised surgical decision-making.

## Supplementary Information

Below is the link to the electronic supplementary material.

Supplementary Material 1

## Data Availability

All data analysed in this study are derived from published articles and are included in the manuscript and supplementary materials.
