# Supplementary material for: Predicting hormonal deterioration following transsphenoidal surgery for non-functioning pituitary adenomas: A systematic review and meta-analysis
Source: Endocrine. 2026 Aug 1;91(1):243. doi: 10.1007/s12020-026-04712-6 (PMC13428792; doi:10.1007/s12020-026-04712-6)
Supplement: Supplementary file 1 — Supplementary Material 1 [file 12020_2026_4712_MOESM1_ESM.docx]

**Supplementary material**

***Search strategy:***

**Databases:**
EBM Reviews - Cochrane Central Register of Controlled Trials <May 2025>
Embase Classic+Embase <1947 to 2025 June 25>
Ovid MEDLINE(R) <1946 to June 25, 2025>

**Table 1 – Search strategy**

| **#** | **Query** | **Results from 26 Jun 2025** |
| --- | --- | --- |
| 1 | pituitary adenoma.mp. or Pituitary Neoplasms/ | 64,665 |
| 2 | NFPA.mp. | 1,518 |
| 3 | nonfunction*.mp. | 31,681 |
| 4 | non-function*.mp. | 31,637 |
| 5 | non functioning pituitary adenoma.mp. | 771 |
| 6 | non-secret*.mp. | 5,430 |
| 7 | (NFA or non functioning adenoma*).mp. [mp=ti, ot, ab, fx, sh, hw, kw, tn, dm, mf, dv, kf, dq, bt, nm, ox, px, rx, ui, sy, ux, mx] | 3,008 |
| 8 | pituitary surgery.mp. or Neurosurgical Procedures/ | 132,559 |
| 9 | Endoscopy/ or transphenoidal.mp. | 224,467 |
| 10 | endonasal.mp. | 18,666 |
| 11 | hypophysectomy.mp. or Hypophysectomy/ | 32,022 |
| 12 | endoscopic endonasal.mp. | 10,484 |
| 13 | hormone*.mp. | 1,894,661 |
| 14 | Treatment Outcome/ or outcome*.mp. | 9,354,605 |
| 15 | hypopituitarism.mp. or Hypopituitarism/ | 30,271 |
| 16 | pituitary function.mp. | 10,207 |
| 17 | endocrine outcome*.mp. | 557 |
| 18 | predict*.mp. | 5,484,615 |
| 19 | Risk/ or Risk Factors/ or risk.mp. | 9,205,617 |
| 20 | prognosis.mp. or Prognosis/ | 2,390,925 |
| 21 | 1 or 2 or 3 or 4 or 5 or 6 or 7 | 126,394 |
| 22 | 8 or 9 or 10 or 11 or 12 | 396,929 |
| 23 | 21 and 22 | 11,740 |
| 24 | 13 or 14 or 15 or 16 or 17 | 11,057,426 |
| 25 | 18 or 19 or 20 | 14,447,427 |
| 26 | 23 and 24 and 25 | 2,045 |
| 27 | remove duplicates from 26 | 1,674 |
| 28 | remove duplicates from 26 | 1,674 |
| 29 | limit 28 to english language | 1,582 |
| 30 | limit 29 to human [Limit not valid in CCTR,Ovid MEDLINE(R); records were retained] | 1,557 |
| 31 | limit 30 to yr="2000 -Current" | 1,477 |

**Supplementary Table 2 -** JBI Risk of bias critical appraisal checklist for Cohort studies

| **JBI Critical Appraisal Checklist for cohort studies** | | | | | | | | | | | |
| --- | --- | --- | --- | --- | --- | --- | --- | --- | --- | --- | --- |
| Questions | Araujo-Castro et al., 2022 | Biamonte et al., 2021 | Chinezu et al., 2017 | Gondim et al., 2015 | Jahangiri et al., 2016 | Little et al., 2020 (a) | Little et al., 2020 (b) | Palpan Flores et la., 2023 | Seejore et al., 2021 | Song et al., 2022 | Vivancos Sanchez et al., 2021 |
| 1.Were the two groups similar and recruited from the same population? | Not applicable | yes | yes | yes | Not applicable | Not applicable | Not applicable | yes | yes | yes | Not applicable |
| 2.Were the exposures measured similarly to assign people to both exposed and unexposed groups? | Not applicable | yes | yes | yes | Not applicable | Not applicable | Not applicable | yes | yes | yes | Not applicable |
| 3. Was the exposure measured in a valid and reliable way? | yes | yes | yes | yes | yes | yes | yes | yes | yes | yes | yes |
| 4.Were confounding factors identified? | yes | yes | Yes | yes | yes | No | No | Yes | yes | yes | yes |
| 5.Were strategies to deal with confounding factors stated? | yes | yes | No | No | yes | No | No | No | yes | No | Yes |
| 6.Were the groups/participants free of the outcome at the start of the study (or at the moment of exposure)? | No | No | No | No | No | No | No | No | No | No | No |
| 7.Were the outcomes measured in a valid and reliable way? | yes | yes | yes | yes | yes | yes | yes | yes | yes | Yes | yes |
| 8.Was the follow up time reported and sufficient to be long enough for outcomes to occur? | yes | yes | yes | yes | yes | yes | yes | yes | yes | Yes | Yes |
| 9.Was follow up complete, and if not, were the reasons to loss to follow up described and explored? | unclear | Unclear | unclear | yes | yes | Unclear | yes | Unclear | Unclear | Unclear | Unclear |
| 10.Were strategies to address incomplete follow up utilized? | unclear | Unclear | Unclear | yes | Unclear | Unclear | Not applicable | Unclear | Unclear | Unclear | Unclear |
| 11.Was appropriate statistical analysis used? | Yes | Yes | Yes | yes | yes | Yes | Yes | Yes | Yes | Yes | Yes |
